# Postoperative Denosumab Therapy in Aneurysmal Bone Cysts and Osteoblastomas: A Case Series and Review of Current Literature

**DOI:** 10.7759/cureus.83997

**Published:** 2025-05-12

**Authors:** Arın Celayir, Erdem Sahin, Mahmut K Ozsahin, Huseyin Botanlioglu

**Affiliations:** 1 Department of Orthopaedics and Traumatology, Cerrahpaşa Faculty of Medicine, Istanbul University-Cerrahpaşa, Istanbul, TUR

**Keywords:** aneurysmal bone cyst, denosumab, osteoblastoma, postoperative therapy, recurrence prevention

## Abstract

Denosumab, a monoclonal antibody targeting receptor activator of nuclear factor-kappa B ligand (RANKL), has demonstrated efficacy in giant cell tumors of bone and has been increasingly explored as a treatment option for aneurysmal bone cysts (ABCs) and osteoblastomas. While its use as a neoadjuvant therapy has been documented, its postoperative role remains unclear. This study presents two cases in which denosumab was administered after surgical resection to evaluate its potential for recurrence prevention and bone remodeling.

The first patient, a 36-year-old female, was diagnosed with a spinal ABC at the T11 level. She underwent curettage, corpectomy, and anterior-posterior spinal instrumentation. Postoperatively, she received denosumab at 120 mg weekly for the first month, followed by 120 mg monthly. At the one-year follow-up, there was no evidence of recurrence, metastasis, or tumor regrowth. The second patient, a 21-year-old woman diagnosed with pelvic osteoblastoma in the left sacrum, underwent curettage and reconstruction with iliac fixation. Approximately 15 months after surgery, she began denosumab therapy, receiving a total of 10 doses, with three doses in the first month (on days 1, 15, and 28), followed by one dose per month at 120 mg. After three years of follow-up, she remained free of recurrence or metastasis, suggesting possible long-term disease control.

These cases highlight the potential role of postoperative denosumab therapy in preventing recurrence and supporting bone remodeling following surgical treatment of ABCs and osteoblastomas. While both patients remained disease-free, the lack of standardized protocols for postoperative denosumab use remains a significant limitation. The optimal timing, dosage, and duration of therapy are yet to be determined. Furthermore, concerns regarding potential complications, such as rebound hypercalcemia upon discontinuation, warrant further investigation. Long-term follow-up and larger studies are necessary to establish clear guidelines and determine whether denosumab can be a reliable adjuvant therapy in the postoperative management of these rare bone tumors.

## Introduction

Aneurysmal bone cysts (ABCs) and osteoblastomas are rare, benign, yet locally aggressive bone lesions that pose considerable clinical challenges, particularly when located in surgically complex regions such as the spine and pelvis [1]. Although surgical resection remains the mainstay of treatment, complete excision may be infeasible or risky in anatomically constrained sites, leading to concerns regarding recurrence and postoperative morbidity.

Denosumab, a fully human monoclonal antibody that inhibits osteoclast-mediated bone resorption by targeting the receptor activator of nuclear factor-kappa B ligand (RANKL) pathway, has emerged as a promising adjunctive therapy for a spectrum of giant-cell-rich bone tumors. While its efficacy is well established in giant-cell tumor of bone (GCTB), accumulating evidence has extended its use to ABCs and osteoblastomas due to their shared histopathological features, including the presence of osteoclast-like giant cells [2].

In ABCs, denosumab has been associated with reduced tumor size, decreased pain, increased ossification, and stabilization of bone structure, although recurrence after discontinuation-particularly in adults-remains a concern [3]. In osteoblastomas, especially those involving the axial skeleton, both pre- and postoperative denosumab use has been explored to facilitate surgical resection, enhance mineralization, and reduce recurrence risk [4]. However, potential complications such as rebound hypercalcemia-particularly in pediatric patients-and histological alterations that may confound pathological evaluation necessitate careful patient monitoring and treatment planning [5].

In this case series, we present two patients: one with a spinal ABC and the other with a pelvic osteoblastoma, both treated with postoperative denosumab. We aim to explore its role in recurrence prevention and bone remodeling while highlighting key clinical considerations.

## Case presentation

Case number 1

**Figure 1:**
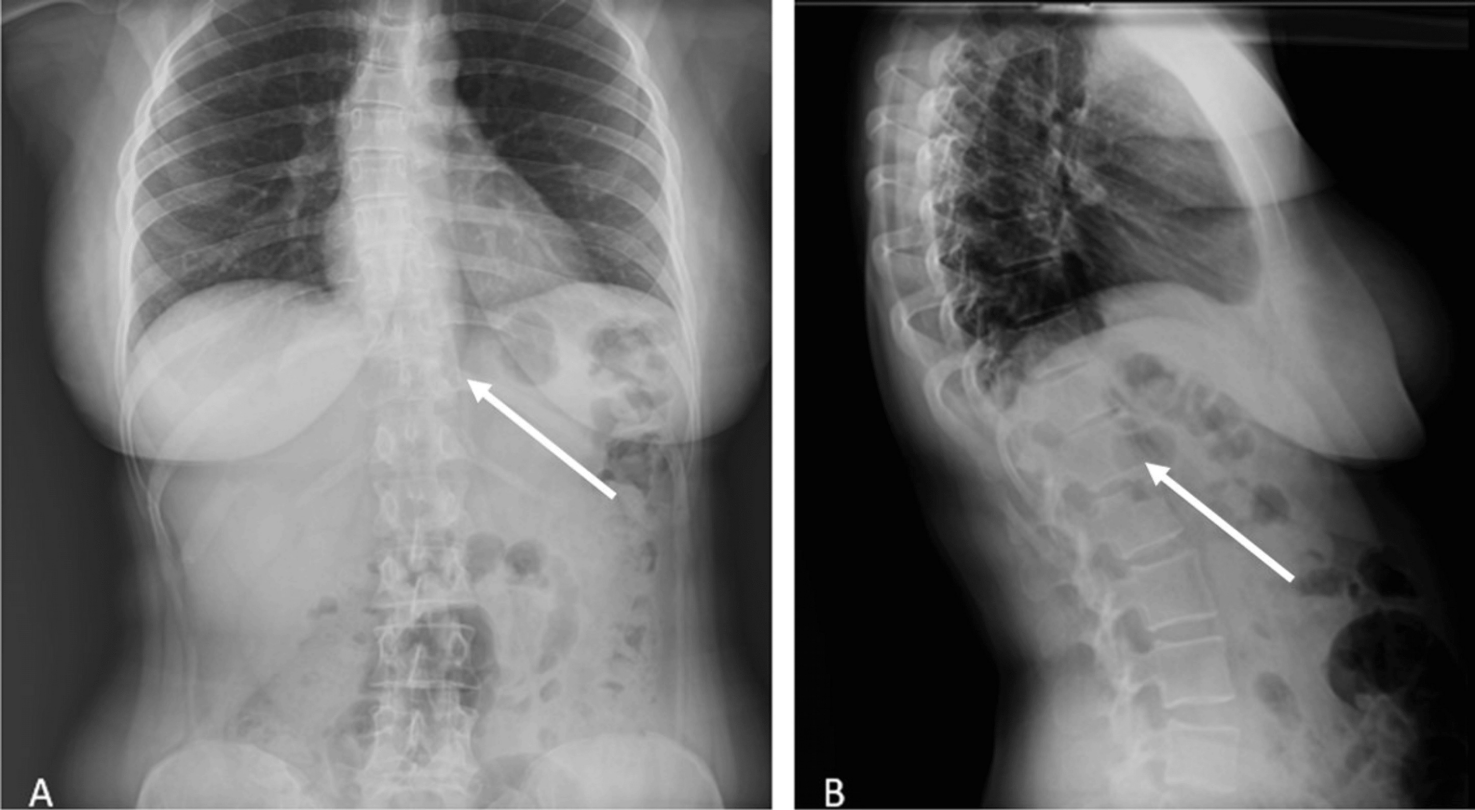
AP and lateral X-ray showing the lesion: (A) scoliosis AP X-ray images of the patient; (B) scoliosis lateral X-ray images of the patient. The white arrows indicate the lesion. AP: anteroposterior

**Figure 2:**
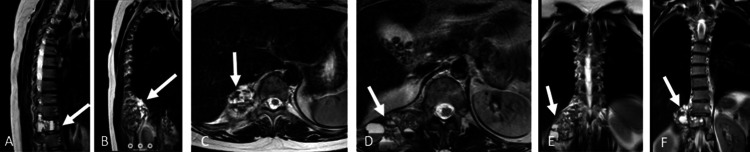
The patient's spinal MRI images: (A and B) sagittal MRI images showing a mass at the T11 level; (C and D) axial MRI images demonstrating the extension of the mass into the paravertebral region on the left side; (E and F) coronal MRI images of the patient demonstrating the mass at the T11 region. White arrows indicate the lesion. MRI: magnetic resonance imaging

A 36-year-old female patient presented to our clinic with complaints of lower back pain. She had no known comorbidities or history of previous surgeries. Informed consent was obtained prior to any intervention. Radiological evaluations, including plain radiographs, magnetic resonance imaging (MRI), and computed tomography (CT), revealed an ABC located at the T11 vertebral level (Figures 1, 2).

A positron emission tomography-CT (PET-CT) scan was also performed, which showed no evidence of metastasis. An initial tru-cut biopsy confirmed the diagnosis of an ABC. Following multidisciplinary discussion at the tumor board, the definitive treatment plan included curettage, bone grafting, corpectomy, and anteroposterior (AP) spinal instrumentation.

**Figure 3:**
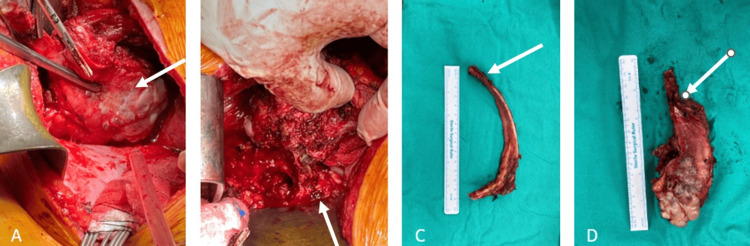
The patient's intraoperative clinical images: (A and B) thoracotomy performed through the 8th intercostal space, followed by resection of the 10th rib, exposing the lesion; (C) the resected 10th rib was prepared for use as a graft; (D) postresection clinical image of the aneurysmal bone cyst. The white arrows in (A and B) indicate the lesion. In (C), the white arrow points to the 11th rib. In (D), it indicates the excised lesion.

The patient subsequently underwent curettage, bone grafting, corpectomy, and AP spinal instrumentation for the ABC at the T11 vertebral level. The procedure was initiated with a thoracotomy through the eighth intercostal space. The 10th rib was resected and prepared for use as an autologous bone graft (Figure 3).

**Figure 4:**
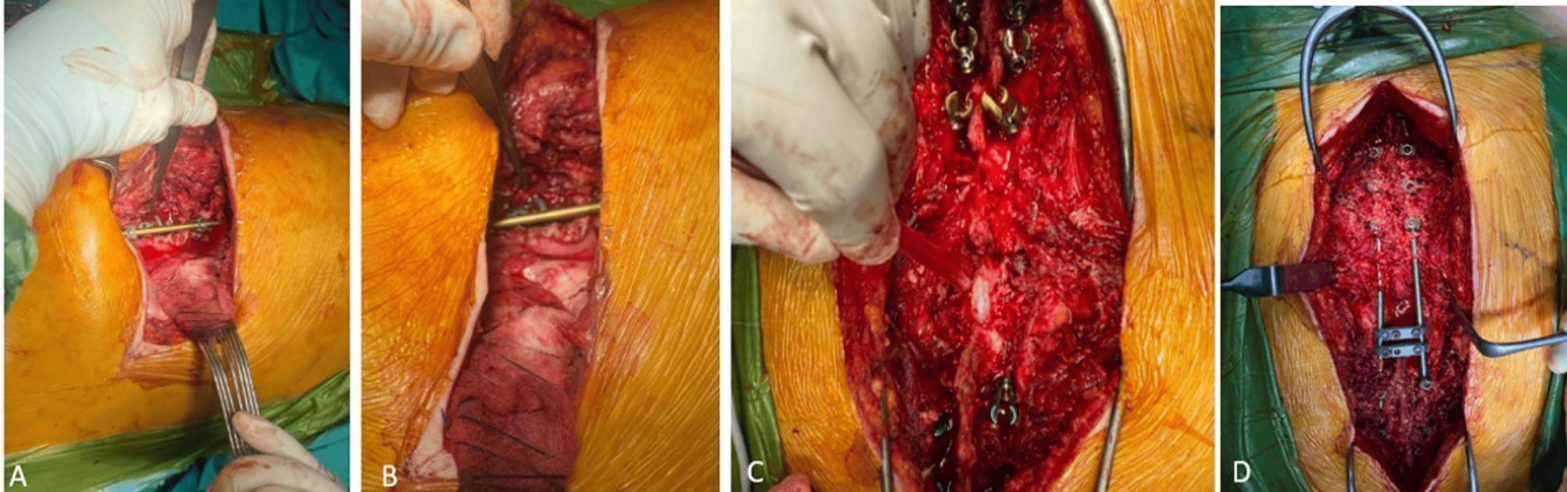
Intraoperative clinical images: (A and B) anterior fixation was achieved with screws and a rod between T10 and T12; (C) the patient was repositioned for posterior instrumentation; (D) pedicle screws were placed from T7 to L2, connected with rods, and further stabilized using transverse connectors.

The ABC was identified around the 11th rib and T11 vertebra. The lesion was curetted, and a corpectomy was performed to create space for cage placement filled with an allograft and rib graft. Anterior instrumentation was completed with screws and a rod fixation between T10 and T12 vertebrae. The patient was then repositioned for posterior instrumentation, where pedicle screws were placed from T7 to L2, connected with rods, and further stabilized with transverse connectors (Figure 4).

**Figure 5:**
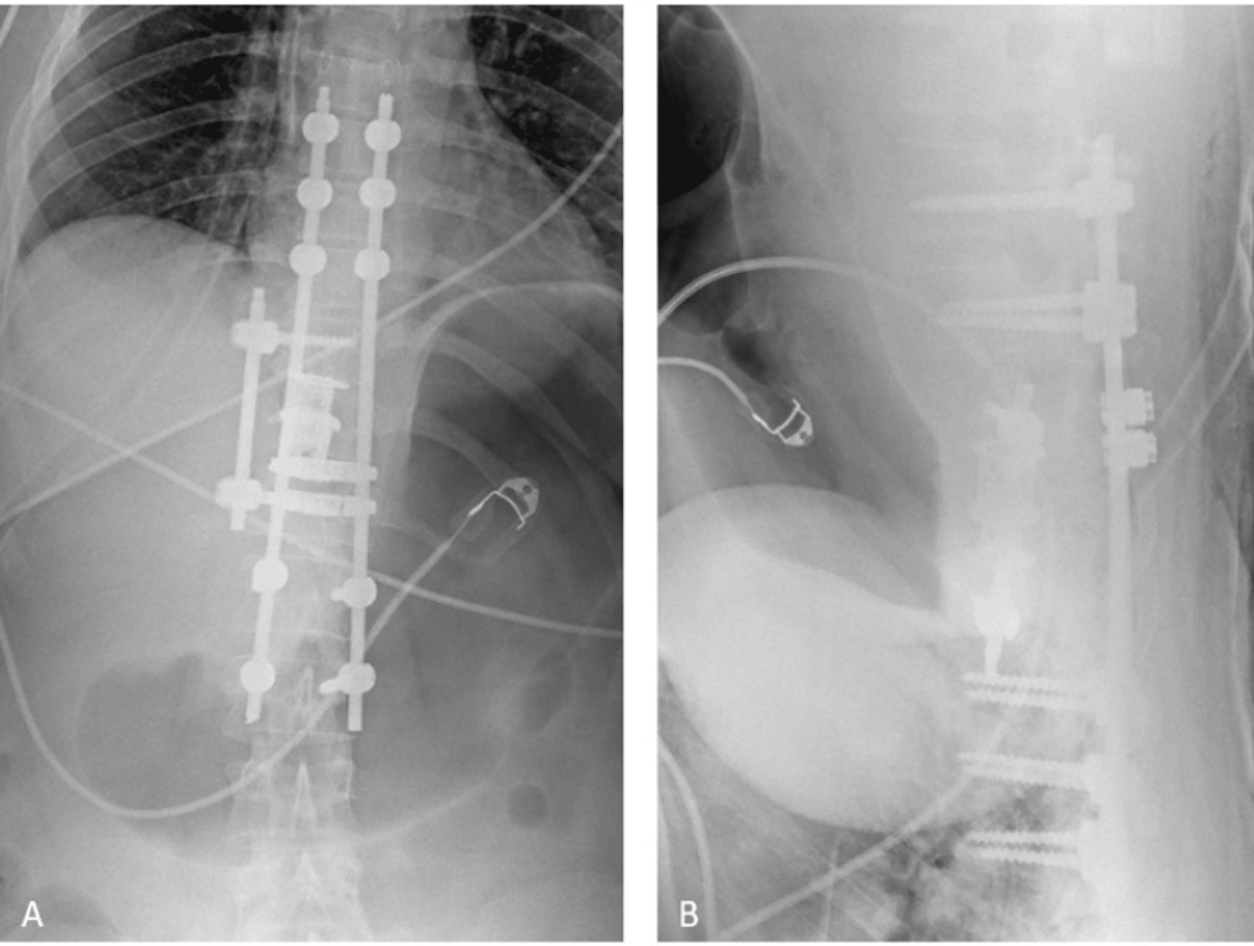
Postoperative radiological images: (A) spinal anteroposterior (AP) view; (B) spinal lateral view.

The procedure was completed with soft tissue closure and neuromonitoring confirmation of spinal integrity. During the postoperative follow-up, the patient showed no signs of wound complications and was discharged (Figure 5).

In the postoperative period, the first patient received a total of five doses of denosumab 120 mg once a week for the first month, followed by monthly dosing, and at the one-year follow-up, no recurrence, metastasis, tumor regrowth, or adverse events such as medication-related osteonecrosis of the jaw (MRONJ) or atypical femoral fractures (AFFs) were observed.

Case number 2

**Figure 6:**
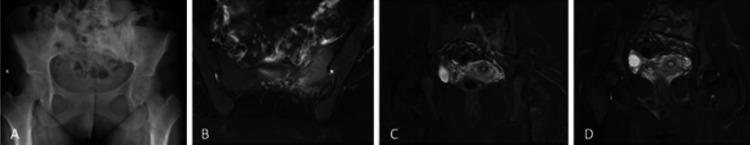
Radiological images: (A) pelvic anteroposterior (AP) view; (B) axial MRI images; (C and D) coronal MRI images. MRI: magnetic resonance imaging

A 21-year-old female patient presented to our clinic with widespread lower back pain. The patient had no known comorbidities and no history of previous surgery and was not on any regular medication. Before any clinical examination, informed consent was obtained. Radiological evaluations revealed a mass in the left sacrum of the pelvis (Figure 6).

An open biopsy performed at an external center was reviewed, and the lesion was diagnosed as an osteoblastoma. MRI measurements showed the tumor size as 43 × 25 × 34 mm, and PET scans revealed no metastasis.

**Figure 7:**
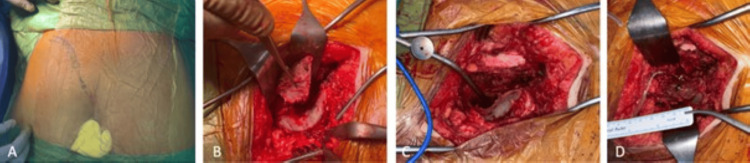
Preoperative clinical images: (A) preoperative marking of the incision, planned as a 12 cm oblique incision from the gluteal region; (B and C) clinical images during and after the V-shaped wedge osteotomy; (D) clinical image after fixation with a cannulated screw.

The patient underwent curettage and reconstruction with iliac fixation for osteoblastoma in the left sacrum. The surgery started with a 12 cm oblique incision from the gluteal region to the posterior iliac spine (Figure 7).

**Figure 8:**
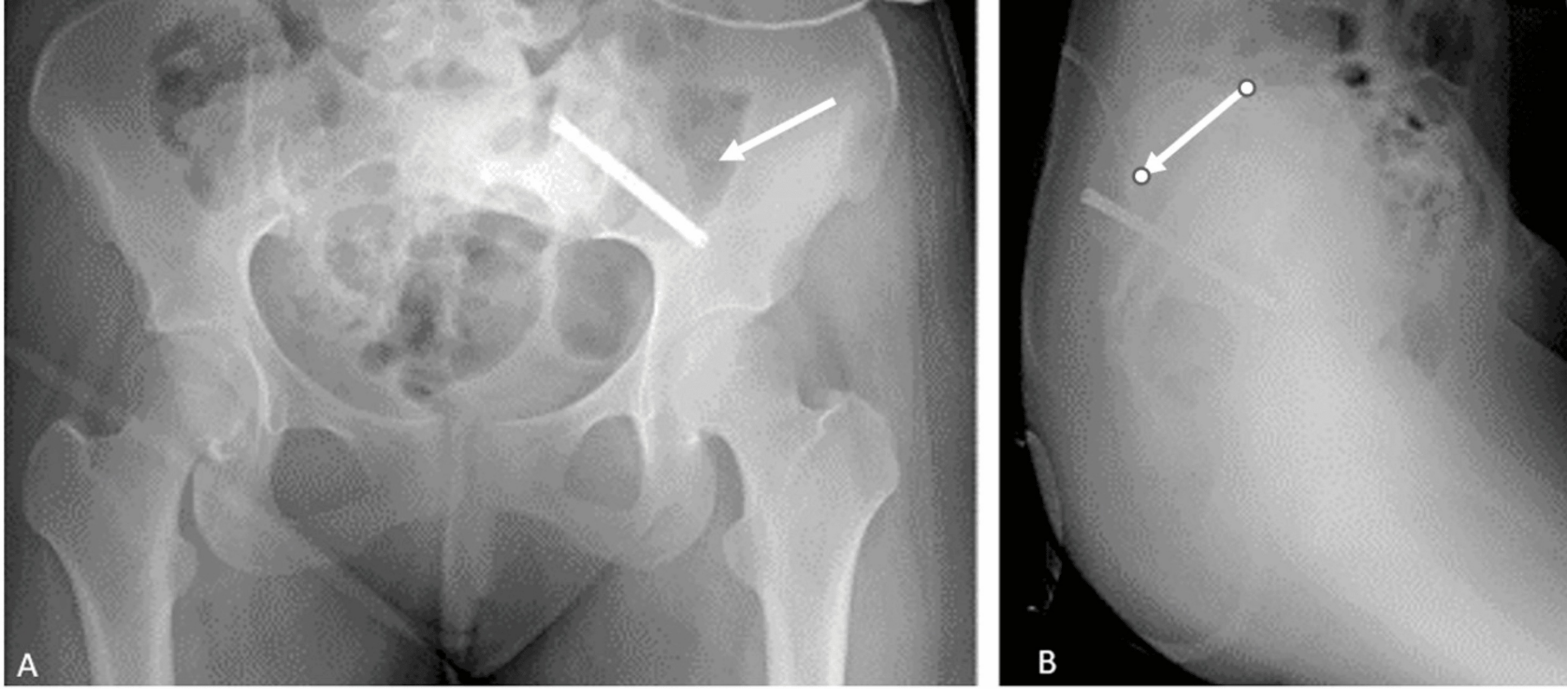
The patient's postoperative radiological images: (A) pelvic anteroposterior (AP) view; (B) coccyx lateral view. The white arrows indicate the postoperative screw.

Paravertebral muscles were dissected to access the ilium and sacrum. A 110 mm cannulated screw was placed in the iliac bone, followed by a V-shaped osteotomy to expose the lesion. Burr-assisted fenestration was performed, and the osteoblastoma was curetted. Phenol-soaked gauze and cauterization were used for hemostasis. Bone wax was applied to the sacrum and iliac bone surfaces, and the excised iliac segment was fixed back with a cannulated screw. The procedure concluded with soft tissue closure and the placement of a Hemovac drain. During the postoperative period, no complications occurred, and the patient was discharged in good general health (Figure 8).

Approximately 15 months after surgery, the patient began denosumab therapy, receiving a total of 10 doses. The treatment started with three doses in the first month (on days 1, 15, and 28), followed by one dose per month thereafter at 120 mg. At the three-year follow-up, no recurrence or metastasis was detected, and no adverse events such as MRONJ or AFF were observed.

## Discussion

The management of ABCs and osteoblastomas remains challenging, particularly in surgically complex locations such as the spine and pelvis. While surgery remains the primary treatment, recurrence and postoperative complications continue to be concerns. In recent years, denosumab, a monoclonal antibody targeting RANKL, has been explored as a potential adjuvant therapy due to its ability to inhibit osteoclast activity, promote tumor mineralization, and reduce the likelihood of recurrence. However, despite promising outcomes in several reports, no standardized protocols have been established for its use after surgery in these conditions, and its role remains largely experimental [6].

Denosumab has demonstrated significant efficacy in managing GCTB, and given the histological similarities between GCTB, ABCs, and osteoblastomas, its off-label use in these tumors has gained interest. Studies suggest that denosumab can induce ossification, reduce pain, and contribute to tumor stabilization, making it particularly valuable in cases where complete surgical excision is challenging. The role of denosumab in osteoblastomas is less well studied, though it has been used preoperatively to shrink tumors and postoperatively to reduce the risk of regrowth.

Our two cases add to the growing but still limited literature on the postoperative use of denosumab in ABCs and osteoblastomas. The first patient with a spinal ABC received denosumab shortly after surgery and showed no recurrence at one year. The second patient with pelvic osteoblastoma began denosumab 15 months postoperatively and remained disease-free at three years, suggesting potential long-term disease stabilization.

These cases provide important real-world insights into postoperative denosumab use in rare bone tumors. Notably, the first case reflects an early initiation strategy, whereas the second case illustrates delayed initiation more than a year after surgery, yet both achieved favorable outcomes. This variability highlights a potential therapeutic window where denosumab may offer benefits regardless of timing, although optimal initiation remains undefined. Moreover, neither patient experienced notable adverse events such as MRONJ, AFF, or rebound hypercalcemia, supporting the potential safety of denosumab when carefully monitored in the postoperative setting. These observations reinforce the need for individualized treatment planning and suggest that denosumab may provide a valuable adjunctive option in cases where surgical margins are compromised or when anatomical constraints limit complete resection.

A recent meta-analysis by Maximen et al. demonstrated denosumab’s effectiveness in reducing tumor size, enhancing ossification, and relieving symptoms in ABCs, particularly in cases where surgery poses high risks (8). However, concerns about recurrence, rebound hypercalcemia, and the absence of standardized protocols remain. Our findings further support its postoperative role, as both of our patients-one with a spinal ABC and the other with a pelvic osteoblastoma-remained recurrence-free after surgery and denosumab therapy. Notably, neither patient experienced rebound hypercalcemia, suggesting that controlled dosing may mitigate this complication. However, our study is limited by a short follow-up period, making it unclear whether denosumab provides long-term tumor control. While our initial results are encouraging, further studies with extended follow-up and larger patient cohorts are essential to establish optimal postoperative protocols for ABCs and osteoblastomas [7].

Denosumab has proven effective in managing GCTB, and its off-label use in ABCs and osteoblastomas is gaining interest due to their histological similarities. Chawla et al. conducted a multicenter phase 2 study on denosumab in GCTB, demonstrating tumor stabilization, ossification, and pain reduction, while also highlighting concerns about recurrence, adverse effects, and rebound hypercalcemia upon discontinuation [8].

Although research on denosumab in ABCs and osteoblastomas is limited, available studies suggest similar benefits in ossification and symptom relief. However, recurrence rates in ABCs reach 18.6% in adults, raising concerns about long-term disease control. The role of denosumab in osteoblastomas is even less understood, though it has been used both preoperatively to shrink tumors and postoperatively to prevent regrowth [9]. While its mechanism of action in all three tumors is similar, denosumab’s regimen for ABCs and osteoblastomas remains unstandardized, unlike in GCTB, where structured guidelines exist. Further studies are needed to define optimal dosing, duration, and long-term safety for its use in these tumors.

One important limitation of our cases is the relatively short follow-up period, which may not be sufficient to fully assess the long-term efficacy of denosumab or the potential for late recurrence. Given that both ABCs and osteoblastomas can recur years after initial treatment, longer follow-up is essential to determine whether denosumab provides sustained disease control or if delayed recurrences may still occur. Further studies with larger patient cohorts and extended observation periods are necessary to establish standardized treatment guidelines and to better define the role of denosumab in the postoperative management of these challenging bone tumors.

## Conclusions

Our case series adds to the limited body of evidence supporting the use of denosumab after surgery for ABCs and osteoblastomas. Both patients remained recurrence-free, suggesting a potential role for denosumab in postoperative management to reduce recurrence risk and promote bone remodeling. Our findings suggest that denosumab may serve as a valuable adjunct in challenging cases where complete surgical resection is difficult or where minimizing morbidity is critical. However, without established treatment guidelines, its use remains experimental, and long-term follow-up and larger studies are needed to determine the ideal patient selection, dosing regimen, and long-term safety profile.

## References

[1] Grahneis F, Klein A, Baur-Melnyk A, Knösel T, Birkenmaier C, Jansson V, Dürr HR (2019). Aneurysmal bone cyst: a review of 65 patients. J Bone Oncol.

[2] Dürr HR, Grahneis F, Baur-Melnyk A, Knösel T, Birkenmaier C, Jansson V, Klein A (2019). Aneurysmal bone cyst: results of an off label treatment with denosumab. BMC Musculoskelet Disord.

[3] Skubitz KM, Peltola JC, Santos ER, Cheng EY (2015). Response of aneurysmal bone cyst to denosumab. Spine (Phila Pa 1976).

[4] Pan KS, Boyce AM (2021). Denosumab treatment for giant cell tumors, aneurysmal bone cysts, and fibrous dysplasia-risks and benefits. Curr Osteoporos Rep.

[5] Lange T, Stehling C, Fröhlich B (2013). Denosumab: a potential new and innovative treatment option for aneurysmal bone cysts. Eur Spine J.

[6] Hung YP, Bredella MA, Lobmaier IV, Lozano-Calderón SA, Rosenberg AE, Nielsen GP (2022). Aneurysmal bone cyst and osteoblastoma after neoadjuvant denosumab: histologic spectrum and potential diagnostic pitfalls. APMIS.

[7] Maximen J, Robin F, Tronchot A, Rossetti A, Ropars M, Guggenbuhl P (2022). Denosumab in the management of aneurysmal bone cyst. Joint Bone Spine.

[8] Chawla S, Blay JY, Rutkowski P (2019). Denosumab in patients with giant-cell tumour of bone: a multicentre, open-label, phase 2 study. Lancet Oncol.

[9] Engellau J, Seeger L, Grimer R (2018). Assessment of denosumab treatment effects and imaging response in patients with giant cell tumor of bone. World J Surg Oncol.

